# Down-regulation of amygdala activation with real-time fMRI neurofeedback in a healthy female sample

**DOI:** 10.3389/fnbeh.2014.00299

**Published:** 2014-09-18

**Authors:** Christian Paret, Rosemarie Kluetsch, Matthias Ruf, Traute Demirakca, Steffen Hoesterey, Gabriele Ende, Christian Schmahl

**Affiliations:** ^1^Department Neuroimaging, Central Institute of Mental Health Mannheim, Medical Faculty Mannheim/Heidelberg UniversityMannheim, Germany; ^2^Department of Psychosomatic Medicine and Psychotherapy, Central Institute of Mental Health Mannheim, Medical Faculty Mannheim/Heidelberg UniversityMannheim, Germany

**Keywords:** affective disorders, amygdala, emotion regulation, mPFC, emotions, real-time fMRI neurofeedback, affective symptoms, instrumental learning

## Abstract

Psychiatric conditions of emotion dysregulation are often characterized by difficulties in regulating the activity of limbic regions such as the amygdala. Real-time functional magnetic resonance imaging (rt-fMRI) allows to feedback brain activation and opens the possibility to establish a neurofeedback (NF) training of amygdala activation, e.g., for subjects suffering from emotion dysregulation. As a first step, we investigated whether feedback of the amygdala response to aversive scenes can improve down-regulation of amygdala activation. One group of healthy female participants received amygdala feedback (*N* = 16) and a control group was presented with feedback from a control region located in the basal ganglia [*N*(sum) = 32]. Subjects completed a one-session rt-fMRI-NF training where they viewed aversive pictures and received continuous visual feedback on brain activation (REGULATE condition). In a control condition, subjects were advised to respond naturally to aversive pictures (VIEW), and a neutral condition served as the non-affective control (NEUTRAL). In an adjacent run, subjects were presented with aversive pictures without feedback to test for transfer effects of learning. In a region of interest (ROI) analysis, the VIEW and the REGULATE conditions were contrasted to estimate brain regulation success. The ROI analysis was complemented by an exploratory analysis of activations at the whole-brain level. Both groups showed down-regulation of the amygdala response during training. Feedback from the amygdala but not from the control region was associated with down-regulation of the right amygdala in the transfer test. The whole-brain analysis did not detect significant group interactions. Results of the group whole-brain analyses are discussed. We present a proof-of-concept study using rt-fMRI-NF for amygdala down-regulation in the presence of aversive scenes. Results are in line with a potential benefit of NF training for amygdala regulation.

## Introduction

The amygdala constitutes a core structure of emotion processing (Phan et al., 2002; Kober et al., 2008). It plays a major role in the generation and modulation of emotional responses in animals (LaBar and LeDoux, 1996) and humans (LaBar et al., 1995; Hermans et al., 2012; Haaker et al., 2013). Accumulating data indicate a close relationship between psychiatric symptoms and an exaggerated amygdala response to emotional material. An excessive response of this structure to emotional information has been shown for borderline personality disorder (BPD) (Niedtfeld et al., 2010; Schulze et al., 2011), depression (Sheline et al., 2001; Victor et al., 2010), phobias (Phan et al., 2006; Goossens et al., 2007), and post-traumatic stress disorder (PTSD) (Fonzo et al., 2010; Simmons et al., 2011)—all of which involve emotional dysregulation. Consequently, improving emotion regulation is one of the central goals of psychotherapy. On the neural level, there is evidence for normalization of the amygdala response with psychotherapy (Sheline et al., 2001; Goossens et al., 2007; Godlewska et al., 2012; Lipka et al., 2013). This is in line with a recent meta-analysis by Buhle et al. (2013), who identified the amygdala as a robust target of cognitive emotion regulation. The decrease of amygdala activation by cognitive reappraisal has been shown to correlate with perceived emotion regulation success (Wager et al., 2008). The neural top-down control of the amygdala is achieved by prefrontal-limbic coupling: specifically, a decrease in amygdala activation is associated with activation in lateral and medial regions of the prefrontal cortex (PFC) (Urry et al., 2006; Wager et al., 2008; Erk et al., 2010; Diekhof et al., 2011). In addition to amygdala hyperactivity, individuals with the aforementioned disorders have previously shown dysfunctional amygdala-prefrontal coupling (Johnstone et al., 2007; Fonzo et al., 2010; Schulze et al., 2011; Simmons et al., 2011; Lang et al., 2012).

Taken together, improving amygdala self-regulation might constitute a pathway to mental health. Hence, there is a need to develop effective therapeutic interventions which could help patients to better control amygdala activation. Several studies have begun to test real-time functional magnetic resonance imaging (rt-fMRI) as a potential therapeutic tool in psychiatric conditions (Linden et al., 2012; Ruiz et al., 2013). With rt-fMRI, a volume of brain data can be processed as soon as it has been scanned. This enables the estimation of the activation in a brain region-of-interest (ROI) for each newly acquired volume. In rt-fMRI neurofeedback (NF), the patient is supplied e.g., with a visual display indicating the current activation level in a ROI. There is increasing evidence that the control of neural circuits of emotion can be achieved by rt-fMRI-NF in healthy individuals as well as psychiatric populations. For example, Young et al. (2014) recently showed that depressed subjects who received feedback from the left amygdala during recall of positive autobiographical memories were able to up-regulate their amygdala response. This corroborates earlier findings in healthy male volunteers (Zotev et al., 2011), who showed a significant Blood Oxygenation Level Dependent (BOLD) signal increase in the left amygdala, which was not found in a control group receiving sham feedback. Another study by Brühl et al. (2014) investigated amygdala down-regulation by rt-fMRI-NF. Specifically, the authors instructed a group of healthy subjects to down-regulate a region in their right amygdala while viewing faces with different emotional expressions. Their results demonstrated an increasing down-regulation effect over the course of four scanning sessions. However, due to the lack of a control group it is not possible to rule out unspecific effects that may have influenced learning, like task repetition or rehearsal of a regulation strategy (Sulzer et al., 2013).

In this proof-of-concept study, we aimed to assess whether subjects can profit from a rt-fMRI-NF training of the amygdala with the aim of down-regulating their amygdala response to aversive scenes, and whether such a training effect can be attributed specifically to having received contingent amygdala feedback. Previous results of our group have shown that the amygdala responds with sustained BOLD activation in aversive picture viewing and identified this region as a potential target region for down-regulation by a rt-fMRI-NF training (Paret et al., 2014). While the reduction of the amygdala response emerges from the literature as one consistent effect of successful emotion regulation (see Diekhof et al., 2011; Buhle et al., 2013 for meta-analyses), the precise prefrontal structures involved in regulation may differ with the cognitive strategy applied (Ochsner et al., 2004; Kalisch et al., 2006; McRae et al., 2010; Kanske et al., 2011; Opialla et al., 2014). We assume, that choosing the signal from the amygdala opposed to a prefrontal region allows for flexibility in strategy-selection. Studies on down-regulation of the amygdala response to aversive material either endorse preferential involvement of the left (Diekhof et al., 2011), right (Brühl et al., 2014), or bilateral (Buhle et al., 2013) amygdala. We combined an anatomical delineation of the bilateral amygdala with a functional voxel selection based on the activation profile during the experiment. This procedure was flexible for including voxels from the right as well as the left amygdala, depending on their activation to the experimental stimuli.

Only female participants were included to control for variance due to potential effects of gender. We chose to elicit emotional activation by presenting aversive pictures which are widely used in the neuroimaging literature of emotion regulation (Kalisch, 2009). A one-session learning protocol was used, closing with a transfer test without NF, where subjects were again presented with aversive pictures and instructed to regulate, but did not receive any feedback. We hypothesized a decrease of the BOLD signal amplitude of the amygdala in blocks where subjects of the experimental group were instructed to regulate vs. view aversive pictures. This finding was expected to be more pronounced in the experimental group compared to a control group receiving feedback from a region located in another part of the brain. Further, we expected the experimental group to show stronger down-regulation of amygdala activation during the transfer run compared to the control group.

Given previous reports of run-to-run improvements during NF training (Zotev et al., 2011; Lawrence et al., 2013), we also evaluated our data for an improvement in amygdala down-regulation over the course of the training.

Veit et al. (2012) have recently used the anterior insula response to aversive pictures as a NF signal for a training of brain self-regulation. The authors found a modulation of the anterior insula response consistent with the instruction to up-regulate and down-regulate a thermometer, which displayed the activation of the region. Together with the amygdala, the anterior insula plays an important role in the processing of emotional information (Damasio et al., 2000; Phan et al., 2002; Kober et al., 2008). To explore the specifity of amygdala NF training on the regulation of brain structures of the affective brain system, we investigated the effect of the training on the activation of the anterior insula.

Finally, we conducted a whole-brain analysis to explore the involvement of other regions.

## Methods

### Sample

*N* = 32 right-handed female participants (*N* = 16 per group) were tested. There were no significant differences in age between the experimental (24.19 ± 4.17, range: 19–34) and the control group (24.94 ± 3.87, range: 20 to 34) [*T*_(30)_ = 0.53]. 14 participants of the experimental group and 15 participants of the control group had a university entrance diploma (German Abitur) (Fisher's Exact Test: *p* = 1.000). Participants reported no current and past DSM-IV Axis I and II disorder or family history of neurological or psychiatric disorders, as confirmed by the Structured Clinical Interview for DSM-IV (SCID; First et al., 1997) as well as the International Personality Disorder Examination (IPDE; Loranger, 1999), which had been conducted before inviting subjects for the experiment. The study was approved by the Ethics Committee of the Medical Faculty Mannheim of the University of Heidelberg, and all subjects provided written informed consent before participation. Compensation for expenses was 24 Euro.

### Group assignment

Subject assignment to groups was randomized and blinded to both the participant and the investigator welcoming and instructing the participant before training. Subjects were informed before the investigation that part of the experiment was to find out which of two regions would be best suited for the training.

### Instruction and visual feedback presentation

Subjects were instructed to regulate “the feeling center” of their brains and were told that this brain region is involved in the “perception and processing of emotions.” No further instructions on the use of a specific regulation strategy were provided. During training, feedback on brain activation was given by the level of a thermometer displayed to both sides of the picture stimulus. The bilateral presentation was chosen to ensure high visibility of the thermometer regardless of gaze orientation. Participants were presented with the following instruction in German language: “If you see the statement “Regulate,” an unpleasant image will be shown. Your task is to regulate the number of bars in the thermometer.” Additionally, subjects were briefed that an orange line in the lower half of the thermometer indicated “the activation of the feeling center under rest or non-emotional conditions” and the aim is “that the bars remain at or below the baseline.” In terms of activation magnitude, one bar of the thermometer display corresponded to 0.2% signal change. The orange line divided the thermometer in an upper part displaying activation (maximum of 2.8% signal change, derived from reported BOLD signal changes in the literature, Zotev et al., 2011 and confirmed by piloting experiments in our group) and a lower part indicating deactivation from baseline (maximum of 1.2% signal change, since we expected more positive signal change compared to baseline in the REGULATE and VIEW conditions, we chose to leave more space for thermometer bars above compared to below). To prevent regulation by just looking at the thermometer or non-emotional details of the picture, participants were advised not to avert their gaze or keep their eyes closed, nor to focus exclusively on the thermometer, but rather to look at the picture for its entire presentation. The participants' eyes were tracked by a camera system (MRC Systems, Heidelberg, Germany) to encourage subjects to adhere to the instructions and to control for drowsiness during training. Data were not statistically analyzed. Subjects were also instructed to consider the temporal latency of the BOLD signal amplitude when evaluating the success of regulating their brain activation.

When a NF run was finished, subjects rated perceived regulation success. They were asked whether they had been able to regulate the thermometer on a 9-point scale (0 = not at all, 9 = very much).

### Experimental conditions

In addition to the “regulate”-condition (REGULATE), the protocol included two control conditions: a “view-negative” (VIEW) and a “view-neutral” (NEUTRAL) condition. Subjects were instructed to refrain from controlling the thermometer during the control conditions. While aversive pictures were presented in VIEW trials, scrambled pictures with no meaningful content were presented during NEUTRAL trials. Trials were separated by an inter-trial interval (ITI) with a fixation cross displayed on the screen.

**Figure 1:**
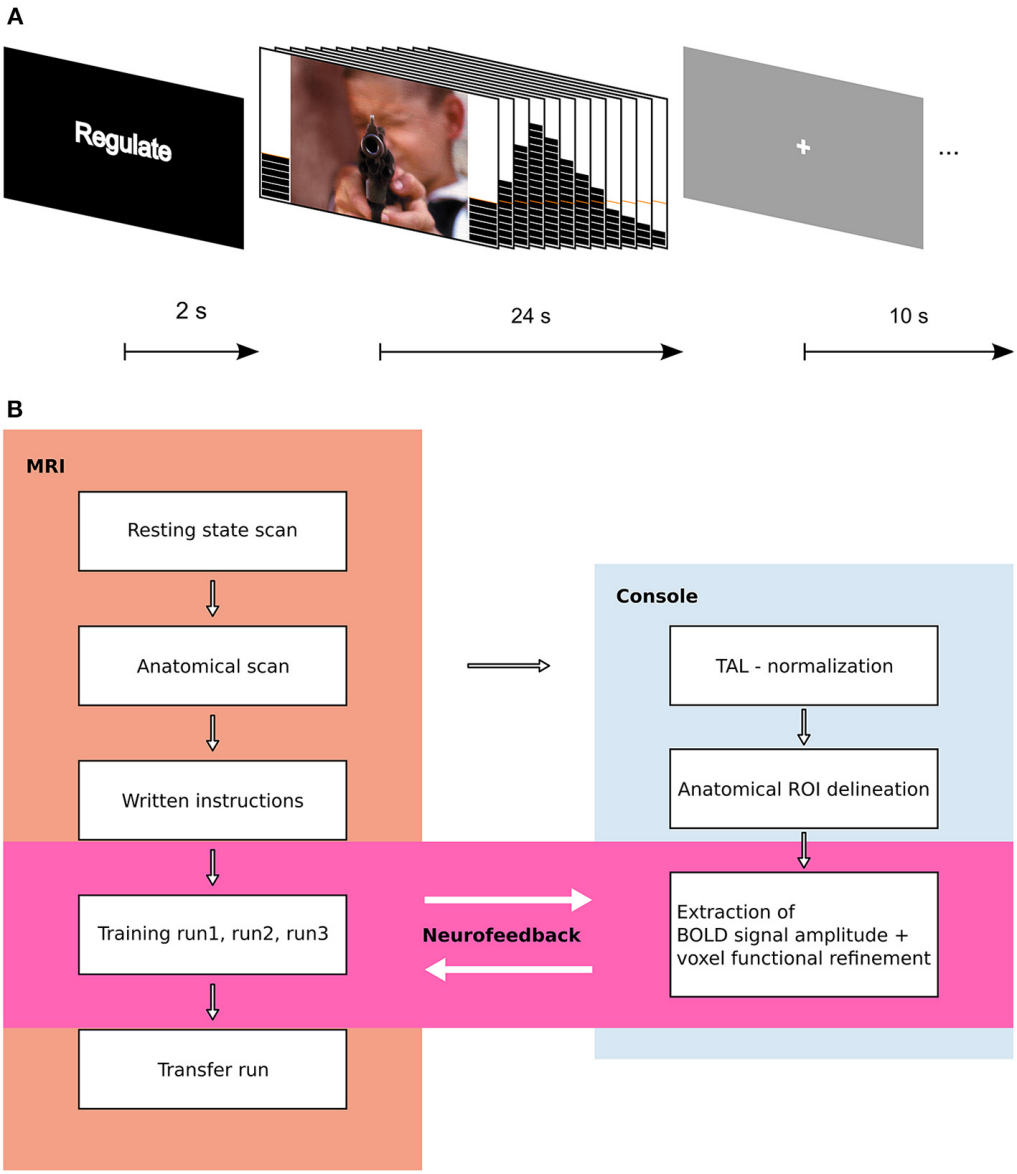
**Experimental design. (A)** Trial structure. A trial started with a 2 s instruction slide indicating trial type (REGULATE, VIEW, NEUTRAL). In the following block, participants either saw an aversive or a scrambled picture with a thermometer at both sides. The thermometer displayed the change in brain activation and was updated every 2 s. After 24 s, the screen changed to gray with a white fixation cross (ITI), and 10 s later, the next trial started. **(B)** Flow Chart of experimental procedure. Results from resting state data are published elsewhere.

The structure of an experimental trial is shown in Figure 1A. An experimental run lasted approximately 9 min and consisted of 15 trials, with 5 of each condition. Each subject participated in 3 consecutive NF training runs, followed by 1 transfer run.

### Stimulus material and presentation

Stimuli were taken from standardized picture series (Lang et al., 2008; Wessa et al., 2010) and were chosen to elicit moderate to high negative valence and arousal. Pictures included in the training runs had a valence level of 2.17 ± 0.49 (mean ± standard deviation (SD), values were taken from the original reports by Lang and colleagues and Wessa and colleagues) and an arousal level of 6.35 ± 0.70. Stimuli included in the transfer run were matched to training stimuli with a valence of 2.12 ± 0.54 and arousal values of 6.26 ± 0.71. Stimuli were assigned to the REGULATE and VIEW conditions matching valence and arousal between runs and conditions (significance value of *p* < 0.05 in a comparison of means) and the stimulus-to-condition assignment was counterbalanced (picture numbers can be obtained from the corresponding author). The condition order was semi-randomized with the restriction of ≤2 consecutive stimuli of the same condition. Each subject of the control group received the same version of the experiment as another subject from the experimental group. An overview on the experimental procedure can be obtained from Figure 1B. Stimuli were presented with Presentation software (Neurobehavioral Systems, Berkeley, CA). After completion of the experiment, subjects rated the pictures outside the MRI suite.

### Delineation of brain area and real-time data processing

**Figure 2:**
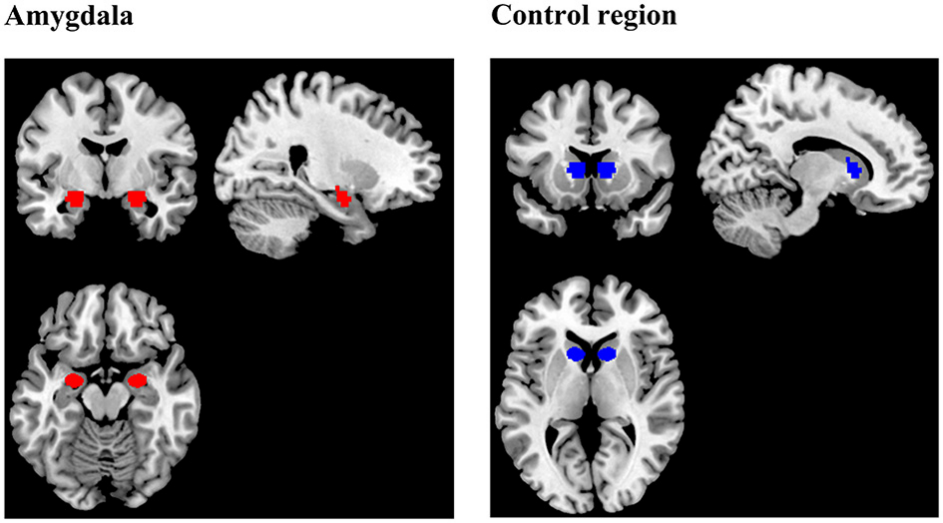
**Regions of interest (ROIs) for the extraction of the neurofeedback signal during training**. The amygdala mask was prepared using the Talairach Daemon (Lancaster et al., 2000) and included voxels which were exclusively assigned to the amygdala by the online tool. The mask delineating the control region was the same size and shape as the amygdala mask, but was located in another part of the brain, comprising parts of rostral basal ganglia, and white matter.

The anatomical scans were imported into BrainVoyager software (version QX2.4, Brain Innovations, Maastricht, Netherlands), skull-stripped and transformed into Talairach space. Normalization parameters were loaded into TurboBrainVoyager (TBV) (version 3.0, Brain Innovations, Maastricht, Netherlands). Depending on group assignment, an anatomical mask of the bilateral amygdala or a mask of a region located in the rostral part of the basal ganglia (control region) was loaded (Figure 2). Due to a technical problem, the Talairach transformation failed in three subjects of the control group. Instead of the control region, these subjects received feedback from a manually drawn square region of variable size (thickness: 5 axial slices), covering parts of the corpus callosum, gray matter, and ventricular areas.

The initial 2 volumes of the functional scans were discarded before real-time processing started. A motion correction feature implemented in TBV was enabled to correct for head movements and spatial smoothing with a 4 mm kernel (full width at half maximum, FWHM) was applied. For the calculation of the BOLD signal amplitude, the “best voxel selection” tool implemented in TBV was used to identify the 33% voxels with beta-values discriminating best between VIEW and NEUTRAL conditions. The voxels were dynamically determined by a score defined by Goebel (2014) based “(a) on the maximum condition beta value and (b) on the amount of deviation from the mean of all condition betas. The first criterion selects those voxels, which have the largest beta value. The second criterion calculates first the mean of all betas and then adds the absolute differences of each beta value from the mean. This deviation index biases the selection to those voxels with a “irregular” profile, i.e., which will show high values for contrasts between betas.” The overall voxel-score is calculated by: voxel-score = (b_max + b_dev)/b_constant. Thereby, the selection of voxels within the spatial region was dynamically refined along the course of training and counterbalanced moderate shifts of the anatomical delineation due to alignment errors across successive runs as well as movement-related slice shifts. Furthermore, the procedure guaranteed that there was no difference in the number of voxels used for signal extraction between subjects and groups. To permit an initial selection of voxels based on their response patterns during VIEW and NEUTRAL, the first two trials of each NF run consisted of those conditions.

The BOLD signal amplitude was passed to Presentation as soon as a new volume had been processed. For each trial, the mean of the last 4 data points before picture onset was taken as a baseline. The signal was smoothed by calculating the mean of the current and the preceding 3 data points. The subtraction of the baseline resulted in the feedback signal amplitude [(X + [X − 1] + [X − 2] + [X − 3])/4-baseline; X = current data point]. The feedback display was updated as soon as information of a new volume had been available. Thus, the latency of the feedback was composed of the TR (2 s) plus the time needed for real-time calculation and display actualization by the presentation software (about half a second).

### Data acquistion and *post-hoc* analysis of imaging data

#### Image acquisition

For brain imaging, a 3 Tesla MRI Scanner (Trio, Siemens Medical Solutions, Erlangen, Germany) with a 32 channel head coil was used. Functional images of the BOLD contrast were acquired with a gradient echo T2^*^ weighted echo-planar-imaging sequence (*TE* = 30 ms, *TR* = 2 s, *FOV* = 192 × 192 mm, flip angle = 80°). One volume comprised 36 slices in AC-PC orientation with a thickness of 3 mm and slice gap of 1 mm. Participants' heads were lightly restrained using soft pads. The four experimental runs comprised 284 volumes each. The T1-weighted anatomical image recording parameters were as follows: *TE* = 3.03 ms, *TR* = 2.3 s, 192 slices and *FOV* = 256 × 256 mm.

#### Preprocessing

FMRI data were analyzed with SPM version 8 (Wellcome Department of Cognitive Neurology, London, UK). Before preprocessing of functional data, nine initial volumes were discarded to avoid T1-equilibrium effects. A slice timing correction of the functional scans was performed with reference to the 18th slice to correct for differences in acquisition time between slices. The functional volumes were spatially aligned to the mean image using a rigid body transformation and images were resliced. Functional images were coregistered to the anatomical image, normalized to the SPM standard template and brought into Montreal Neurological Institute (MNI) coordinate space. Finally, images were smoothed with a kernel of 6 mm (FWHM).

#### Statistical analysis

***First-level analysis***. We formulated separate models in SPM for the NF training and the transfer run. The three NF runs were modeled as separate sessions. Three experimental conditions were modeled (REGULATE, VIEW, NEUTRAL) and the movement vectors taken from the spatial realignment procedure were included in the model as nuisance variables. All events were modeled as blocks of brain activation and were convolved with the hemodynamic response function. Data were high-pass filtered (128 s) and a correction for serial correlations was implemented by autoregressive modeling.

***ROI analysis***. To test our hypotheses, voxel-wise *T*-tests of parameter estimates for the contrasts VIEW>REGULATE, REGULATE>NEUTRAL, and VIEW>NEUTRAL were conducted on the subject level. The mean contrast value was then extracted from all voxels of the amygdala and from the control region. Anatomical templates were used as provided by the Wake Forest University (WFU) PickAtlas toolbox (Maldjian et al., 2003) to delineate the amygdala (left: 71 voxels, right: 76 voxels). To assess results of the control region, we extracted mean contrast values using the control region mask (left and right: 43 voxels each).

Values were screened for outliers before the analysis. As an exclusion criterion, we set a 3 SD threshold below and above the group mean, based on the mean contrast value taken from the bilateral region masks. When outliers were identified, we report test-values with and without outlier exclusion.

Extracted contrast values were passed to SPSS version 20 for statistical analyses.

To test our main hypothesis regarding down-regulation of brain activation (REGULATE) as contrasted with natural viewing of the aversive stimulus (VIEW), a Region (2) × Hemisphere (2) × Group (2) analysis of variance (ANOVA) was calculated for the REGULATE>VIEW contrast. Since the dynamic voxel selection principally allowed the inclusion of more voxels of the one or the other laterality into the ROI, we included the factor of Hemisphere in our analysis and assessed down-regulation for each hemisphere separately.

Where we had directional a-priori hypotheses, one-tailed *t*-tests were calculated to estimate significance (*p* < 0.05). We report a trend when the *p*-value was below *p* = 0.10.

To further characterize group differences and condition-effects, we report results of an additional ANOVA for each region (amygdala, control region; separately for the left and right hemisphere), taking into account the factor Condition (REGULATE>NEUTRAL, VIEW>NEUTRAL).

To explore the effect of NF training on anterior insula regulation, a ROI analysis of parameter estimates was calculated. Mean contrast values were extracted from spherical masks with a radius of 8 mm and centers taken from the literature (left peak: [−33, 20, 0], 82 voxels; right: [36, 26, 6], 81 voxels) (Caria et al., 2007).

***Exploratory whole-brain analysis***. To elucidate task-related effects, we conducted exploratory whole-brain random effects analyses for both groups independently on the t-contrasts VIEW>REGULATE, REGULATE>NEUTRAL and VIEW>NEUTRAL. Additionally we explored group differences, assuming independent measurements and equal variances of errors. To protect against false positives, we used Monte-Carlo simulations to estimate the cluster-extend at a voxel-threshold of *p* < 0.001 and a cluster-threshold of *p* < 0.05. Simulations were performed with 3dClustSim, implemented in AFNI (Cox, 1996). Estimation of cluster-extend was determined in 10.000 iterations, based on the number of voxels included in the masks and the smoothness (FWHM) of the residuals of the second-level SPM8-analyses. The resulting number of voxels (k) is indicated in the results tables.

### Analysis of rating data

Picture-rating data of 2 participants was lost. We looked for group differences in subjective arousal and valence elicited by picture viewing and conducted Group × Condition (VIEW, REGULATE) ANOVAs for pictures presented during training and transfer. To further determine whether groups differed in their perceived regulation success, we conducted a repeated measures ANOVA on regulation success, with “Run” as the within-subjects factor and “Group” as the between-subjects factor.

### Analysis of thermometer variability

To compare the dynamics of the thermometer display between the groups, the number of bars presented to a subject at any given time point in the experiment was derived from the data. The variance of the number of bars as a measure of thermometer variability was calculated for each subject and each condition, and was taken to a Condition (3) × Run (3) × Group (2) ANOVA.

## Results

### ROI analysis

Since three participants of the control group did not receive feedback from the standardized control region due to a technical error, we additionally report results of an analysis leaving out these subjects.

#### Neurofeedback training

**Figure 3:**
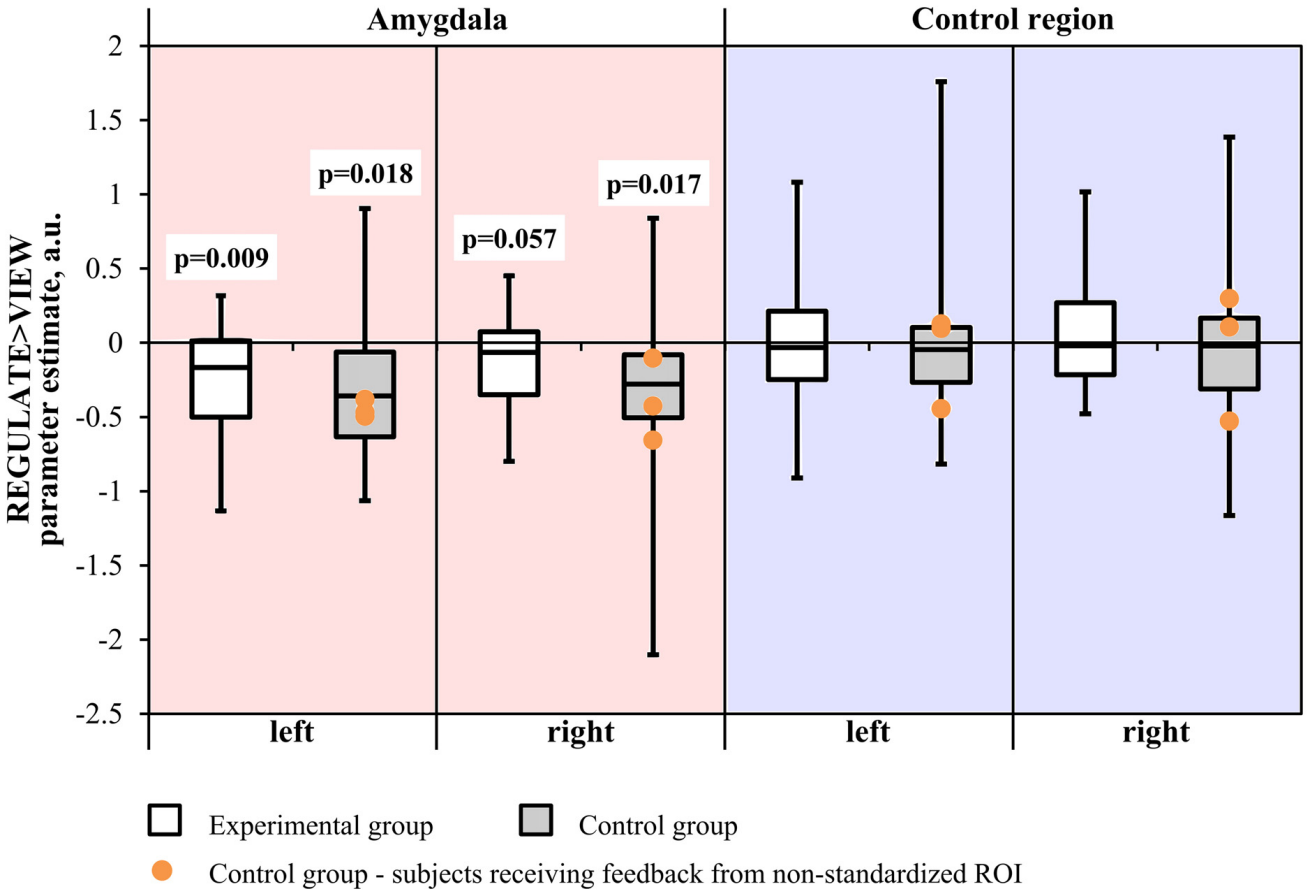
**Results of the region-of-interest analysis of the neurofeedback training**. Negative values indicate a decrease of the BOLD signal amplitude in the regulate condition (REGULATE) compared to natural responding toward aversive pictures (VIEW). White box plots: participants receiving feedback from the amygdala during training (experimental group). Gray box plots: participants receiving feedback from a standardized control region during training (control group). Orange dots: participants who had received feedback from a non-standardized control region during training due to a technical error (*N* = 3). *P*-values indicate probability of the findings for each group and mask under the null-hypothesis. *T*-tests were not significant when correcting for multiple comparisons. A.u., artificial units.

Neither the Region × Hemisphere × Group ANOVA nor the Hemisphere × Group ANOVA of the amygdala revealed significant interactions. The main effect of Region was significant [*F*_(1, 31)_ = 33.163, *p* < 0.001]. Subjects from the experimental group showed down-regulation of the left amygdala in the REGULATE>VIEW contrast [−0.25 ± 0.38 (mean ± *SD*), *T*_(15)_ = 2.675, *p* = 0.009, one-tailed] in the hypothesized direction (Figure 3). The effect was less pronounced in the right amygdala [−0.15 ± 0.34, *T*_(15)_ = 1.684, *p* = 0.057]. The control group showed a similar effect in the left [−0.31 ± 0.53, *T*_(15)_ = 2.324, *p* = 0.018] and right amygdala [−0.36 ± 0.62, *T*_(15)_ = 2.338, *p* = 0.017]. All *t*-tests were not significant when correcting for multiple comparisons.

An exploratory ANOVA of the run-to-run change in the REGULATE>VIEW contrast revealed a trend for a Hemisphere by Group interaction [*F*_(1, 30)_ = 3.164, *p* = 0.085] and a significant main effect of Region [*F*_(1, 30)_ = 33.394, *p* < 0.001]. Since a visual inspection did not suggest a linear trend of the outcome measure in the experimental group, we did not further explore within-group run-to-run changes in parameter estimates.

Excluding subjects who did not receive feedback from the standardized control region did not change significance of the test values.

To further elucidate condition effects, we analyzed the REGULATE>NEUTRAL and VIEW>NEUTRAL contrasts (Table 1). Consistent with the previous results, we detect a significant main effect of Condition in the analysis of the left and right amygdala, while Group-interactions were not significant.

**Table 1:** **Group statistics and ANOVA results of neurofeedback training**.

**(A)**			**Amygdala**										
	**Hemisphere:**		**Left**						**Right**				
	**Run:**		**1**		**2**		**3**		**1**		**2**		**3**
**GROUP STATISTICS (MEAN ± *SD*)**													
Experimental group	REG>NEU		0.23 ± 0.16		0.13 ± 0.27		0.14 ± 0.20		0.21 ± 0.18		0.19 ± 0.27		0.22 ± 0.20
(*N* = 16)	VIEW>NEU		0.35 ± 0.20		0.19 ± 0.23		0.21 ± 0.21		0.30 ± 0.17		0.23 ± 0.22		0.23 ± 0.20
Control group	REG>NEU		0.25 ± 0.21		0.19 ± 0.18		0.11 ± 0.29		0.22 ± 0.22		0.18 ± 0.16		0.15 ± 0.27
(*N* = 16)	VIEW>NEU		0.29 ± 0.13		0.27 ± 0.29		0.29 ± 0.27		0.31 ± 0.22		0.28 ± 0.28		0.32 ± 0.31
**ANOVA RESULTS (*P*-VALUES)**													
Interaction effects	Run × condition × group		0.308						0.271				
	Condition × group		0.747						0.229				
	Run × group		0.628						0.994				
	Run × condition		0.581						0.887				
Main effects	Group		0.578						0.776				
	Run		0.104						0.669				
	Condition		0.002						0.008				
**(B)**		**Control region**											
	**Hemisphere:**	**Left**						**Right**					
	**Run:**	**1**		**2**		**3**		**1**		**2**		**3**	
**GROUP STATISTICS (MEAN ± *SD*)**													
Experimental group	REG>NEU	0.16 ± 0.18		0.12 ± 0.27		0.22 ± 0.18		0.18 ± 0.19		0.15 ± 0.25		0.22 ± 0.19	
(*N* = 16)	VIEW>NEU	0.22 ± 0.16		0.14 ± 0.20		0.17 ± 0.19		0.23 ± 0.19		0.11 ± 0.20		0.15 ± 0.19	
Control group	REG>NEU	0.24 ± 0.33		0.22 ± 0.30		0.10 ± 0.24		0.24 ± 0.32		0.22 ± 0.25		0.13 ± 0.29	
(*N* = 16)	VIEW>NEU	0.21 ± 0.20		0.19 ± 0.38		0.17 ± 0.29		0.20 ± 0.18		0.22 ± 0.34		0.20 ± 0.32	
**ANOVA RESULTS (*P*-VALUES)**													
Interaction effects	Run × condition × group	0.098						0.144					
	Condition × group	0.942						0.642					
	Run × group	0.492						0.592					
	Run × condition	0.942						0.922					
Main effects	Group	0.720						0.623					
	Run	0.714						0.718					
	Condition	0.890						0.866					

*Table shows parameter-estimate-contrast values from the regions of interest. **(A)** Amygdala, **(B)** Control region. SD, standard deviation; REG, regulate; NEU, neutral*.

#### Transfer run

**Figure 4:**
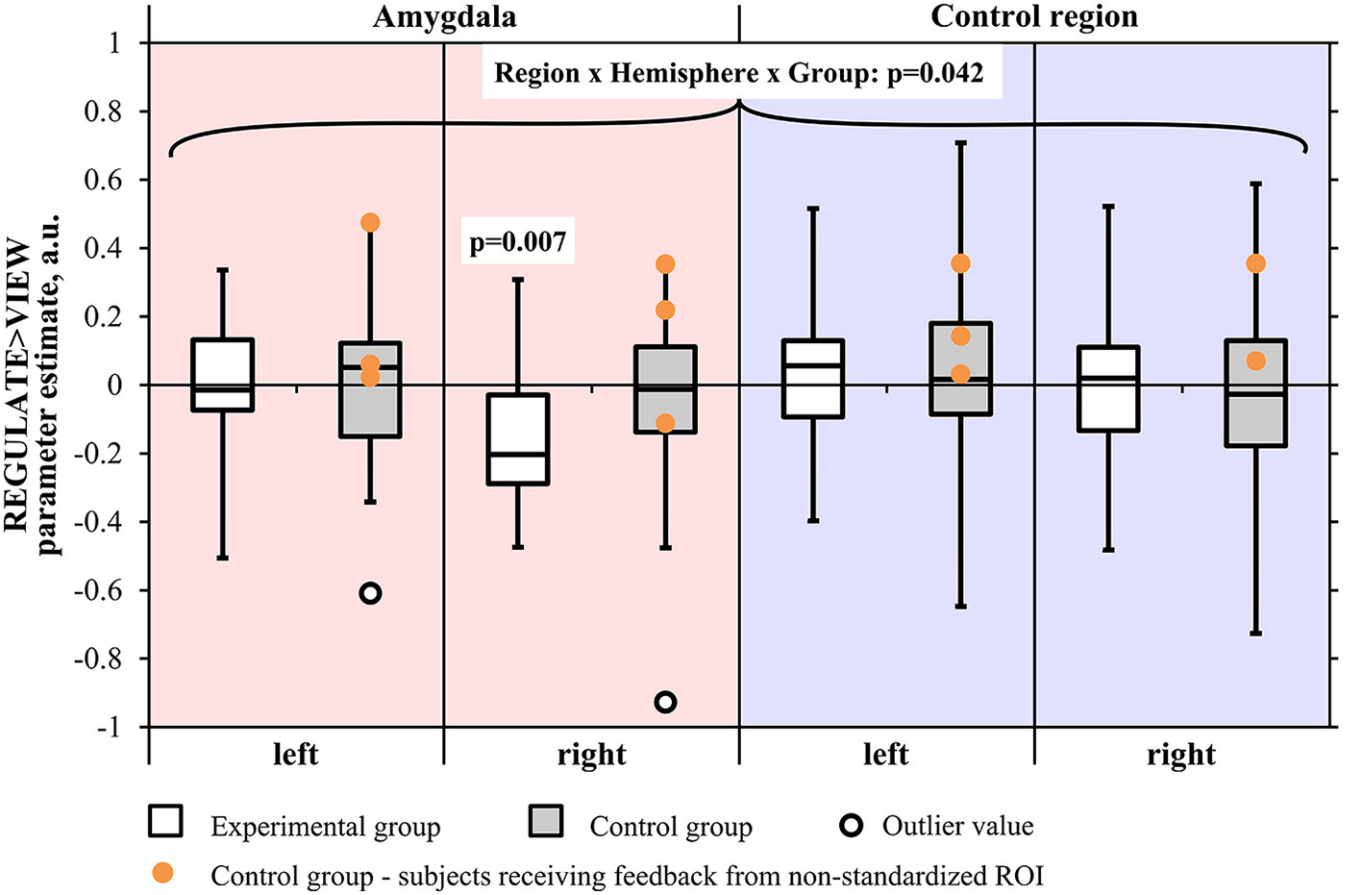
**Results of the region-of-interest analysis of the transfer run**. Negative values indicate a decrease of the BOLD signal amplitude in the regulate condition (REGULATE) compared to natural responding toward aversive pictures (VIEW). White box plots: participants receiving feedback from the amygdala during training (experimental group). Gray box plots: participants receiving feedback from a standardized control region during training (control group). Orange dots: participants who had received feedback from a non-standardized control region during training due to a technical error (*N* = 3). *P*-values indicate probability of the findings for each group and mask under the null-hypothesis. Tests were not significant when correcting for multiple comparisons. Black circle: outlier value (3 standard deviations > group mean). A.u., artificial units.

The screening for outlier values had identified one outlier in the control group (i.e., contrast-value of 3 SD below the group mean). The Region by Hemisphere by Group interaction was significant [*F*_(1, 29)_ = 4.550, *p* < 0.05], with outlier inclusion there was still a trend [*F*_(1, 30)_ = 3.452, *p* = 0.073] (Figure 4). There was a main effect of Region [*F*_(1, 29)_ = 5.172, *p* < 0.05; including the outlier: *F*_(1, 30)_ = 5.701, *p* < 0.05] and Hemisphere [*F*_(1, 29)_ = 19.012, *p* < 0.001, including the outlier: *F*_(1, 30)_ = 21.023, *p* < 0.001]. When excluding subjects who had received feedback from a non-standardized control region, the results did not change in terms of significance regarding the three-way interaction [*F*_(1, 26)_ = 4.618, *p* < 0.05; including the outlier: *F*_(1, 27)_ = 3.339, *p* = 0.079]. The main effects of Region [*F*_(1, 26)_ = 5.675, *p* < 0.05; including the outlier: *F*_(1, 27)_ = 6.326, *p* < 0.05] and Hemisphere [*F*_(1, 26)_ = 18.314, *p* < 0.001; including the outlier: *F*_(1, 27)_ = 20.942, *p* < 0.001] were still significant.

The two-way ANOVA of the amygdala proved the Hemisphere by Group interaction to be at trend [*F*_(1, 29)_ = 3.921, *p* = 0.057; including the outlier: *F*_(1, 30)_ = 2.526, *p* = 0.122] with a significant main effect of Hemisphere [*F*_(1, 29)_ = 10.246, *p* < 0.01; including the outlier: *F*_(1, 30)_ = 11.922, *p* < 0.01]. When excluding subjects receiving feedback from a non-standardized control region, the trend regarding the interaction was robust [*F*_(1, 26)_ = 3.435, *p* = 0.075; including the outlier: *F*_(1, 27)_ = 2.016, *p* = 0.167] as was the significance of the main effect of Hemisphere [*F*_(1, 26)_ = 8.934, *p* < 0.01; including the outlier: *F*_(1, 27)_ = 10.944, *p* < 0.01].

An inspection of subject means suggested a right-lateralized effect. We conducted a Region × Group ANOVA of the values from the right hemisphere masks. The Region by Group interaction was found at trend level [*F*_(1, 29)_ = 3.491, *p* = 0.072; including the outlier: *F*_(1, 30)_ = 3.046, *p* = 0.091] and the main effect of Region was significant [*F*_(1, 29)_ = 7.765, *p* < 0.01; including the outlier: *F*_(1, 30)_ = 9.028, *p* < 0.01], also when excluding subjects who had received feedback from a non-standardized control region [Region by Group interaction: *F*_(1, 26)_ = 2.956, *p* = 0.097; including the outlier: *F*_(1, 27)_ = 2.500, *p* = 0.125; main effect of Hemisphere: *F*_(1, 26)_ = 7.468, *p* < 0.05; including the outlier: *F*_(1, 27)_ = 8.964, *p* < 0.01]. The Region × Group ANOVA did not indicate a significant interaction effect in the left hemisphere.

When contrasting groups, we observed a trend for lower values in the experimental group compared to the control group in the right amygdala [*T*_(29)_ = 1.522, *p* = 0.070, one-tailed; including the outlier subject: *T*_(30)_ = 0.648, *p* = 0.261, one-tailed]. Contrast estimates in the left amygdala did not differ between groups. When excluding subjects who had received feedback from a non-standardized control region, the group difference in the right amygdala was not anymore at trend level [*T*_(26)_ = 0.921, *p* = 0.183; including the outlier subject: *T*_(27)_ = 0.062, *p* = 0.476]. In the control region, we did not find any significant group differences [left: *T*_(29)_ = 0.391, *p* = 0.698, right: *T*_(29)_ = 0.020, *p* = 0.985; including outlier: left: *T*_(30)_ = 0.073, *p* = 0.942, right: *T*_(30)_ = 0.470, *p* = 0.642].

**Figure 5:**
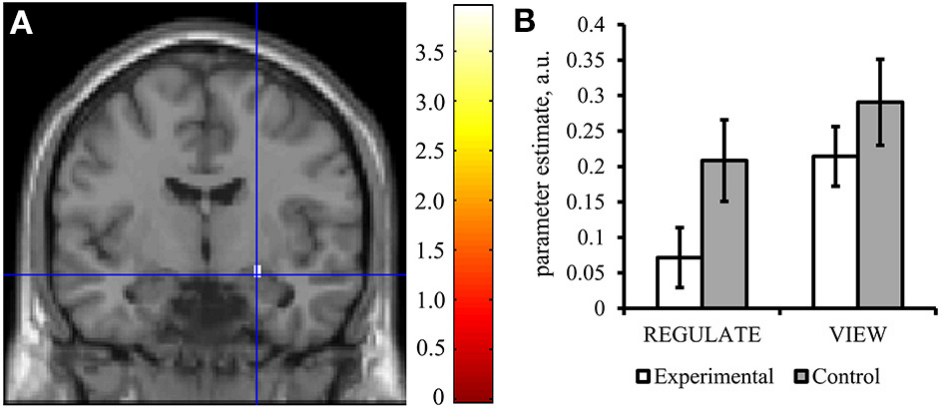
**The experimental group down-regulated the right amygdala response in the transfer run. (A)** Voxels were found activated at *p* < 0.05 (family-wise error corrected for multiple comparisons) in an anatomical mask of the right amygdala (Tzourio-Mazoyer et al., 2002) for the contrast VIEW>REGULATE in the experimental group. Crosshairs indicate location of the peak voxel at [24, −7, −14], Montreal Neurological Institute coordinate space. Activation is displayed on a coronal view of the SPM8 canonical standard template. Left is left. Scale indicates *t*-values of the parameter estimate. **(B)** Mean parameter estimate of all voxels within the right amygdala mask of the REGULATE>NEUTRAL and VIEW>NEUTRAL contrast, taken from the transfer run. White bars: experimental group (*N* = 16), gray bars: control group (*N* = 16). Error bars indicate standard error of mean. A.u., artificial units.

The experimental group showed down-regulation of the right amygdala [REGULATE>VIEW contrast: −0.14 ± 0.20, *T*_(15)_ = 2.797, *p* = 0.007, one-tailed] (Figure 5A) but not the left amygdala [−0.01 ± 0.20, *T*_(15)_ = 0.178, *p* = 0. 861, two-tailed]. The control group did not show down-regulation in the left [0.01 ± 0.22, *T*_(15)_ = 0.099, *p* = 0.923, two-tailed; including outlier subject: −0.03 ± 0.26, *T*_(15)_ = 0.506, *p* = 0.310, one-tailed] and right amygdala [−0.03 ± 0.22, *T*_(14)_ = 0.451, *p* = 0.330, one-tailed; including the outlier subject: −0.08 ± 0.31, *T*_(15)_ = 1.056, *p* = 0.154, one-tailed]. However, all *t*-tests were not significant after correcting for multiple comparisons.

Results didn't change in terms of significance when excluding subjects from the analysis who had received feedback from a non-standardized ROI [left amygdala: −0.07 ± 0.20, *T*_(11)_ = 0.708, *p* = 0.247; including the outlier subject: −0.08 ± 0.24, *T*_(12)_ = 1.235, *p* = 0.120, one-tailed; right amygdala: −0.14 ± 0.31, *T*_(12)_ = 1.602, *p* = 0.068, including outlier subject: −0.07 ± 0.20, *T*_(11)_ = 1.200, *p* = 0.127].

We examined training effects on activation of the control region and did not find significant regulation in the REGULATE>VIEW contrast in both groups, as well as no group-differences of the contrast values.

The results of the Condition × Group ANOVA can be obtained from Table 2. Descriptively, subjects of the experimental group showed lower activations in both REGULATE and VIEW trials compared to the control group (Figure 5B). In the right amygdala, the main effect of Group showed a trend and the main effect of Condition was significant, however, the Condition × Group interaction was not significant. *Post-hoc t*-tests of group differences in the single conditions brought a trend for the experimental group showing lower values than the control group when instructed to regulate. In line with the results presented above, the experimental group and not the control group did show a significant reduction of the right amygdala response in the REGULATE vs. the VIEW condition in the transfer run. Regarding the control region, we detected a significant effect of Group, with participants of the control group showing higher values than the experimental group during both conditions.

**Table 2:** **Group statistics and ANOVA results of the transfer run**.

	**Hemisphere:**	**Amygdala**		**Control region**	
		**Left**	**Right**	**Left**	**Right**
**GROUP STATISTICS (MEAN ± *SD*)**					
Experimental group	REG>NEU	0.19 ± 0.04	0.07 ± 0.04	0.02 ± 0.06	0.00 ± 0.06
(*N* = 16)	VIEW>NEU	0.20 ± 0.05	0.21 ± 0.04	−0.02 ± 0.04	−0.01 ± 0.04
Control group	REG>NEU	0.20 ± 0.07	0.21 ± 0.06	0.20 ± 0.06	0.18 ± 0.05
(*N* = 16)	VIEW>NEU	0.24 ± 0.05	0.29 ± 0.06	0.18 ± 0.06	0.22 ± 0.06
**ANOVA RESULTS (*P*-VALUE)**					
Interaction effect	Condition × group	0.774	0.522	0.823	0.614
Main effects	Group	0.677	0.067	0.003	0.003
	Condition	0.611	0.022	0.595	0.798
*Post-hoc t*-test (*p*-value, two-tailed)	REG>NEU: control vs. experimental	0.845	0.067	0.042	0.045
	VIEW>NEU: control vs. experimental	0.600	0.308	0.011	0.007
	Experimental group: REG vs. VIEW	0.861	0.014	0.540	0.842
	Control group: REG vs. VIEW	0.620	0.308	0.847	0.632

*Table shows parameter-estimate-contrast values from the regions of interest. SD, standard deviation; REG, regulate; NEU, neutral*.

#### Anterior insula: neurofeedback training and transfer run

**Figure 6:**
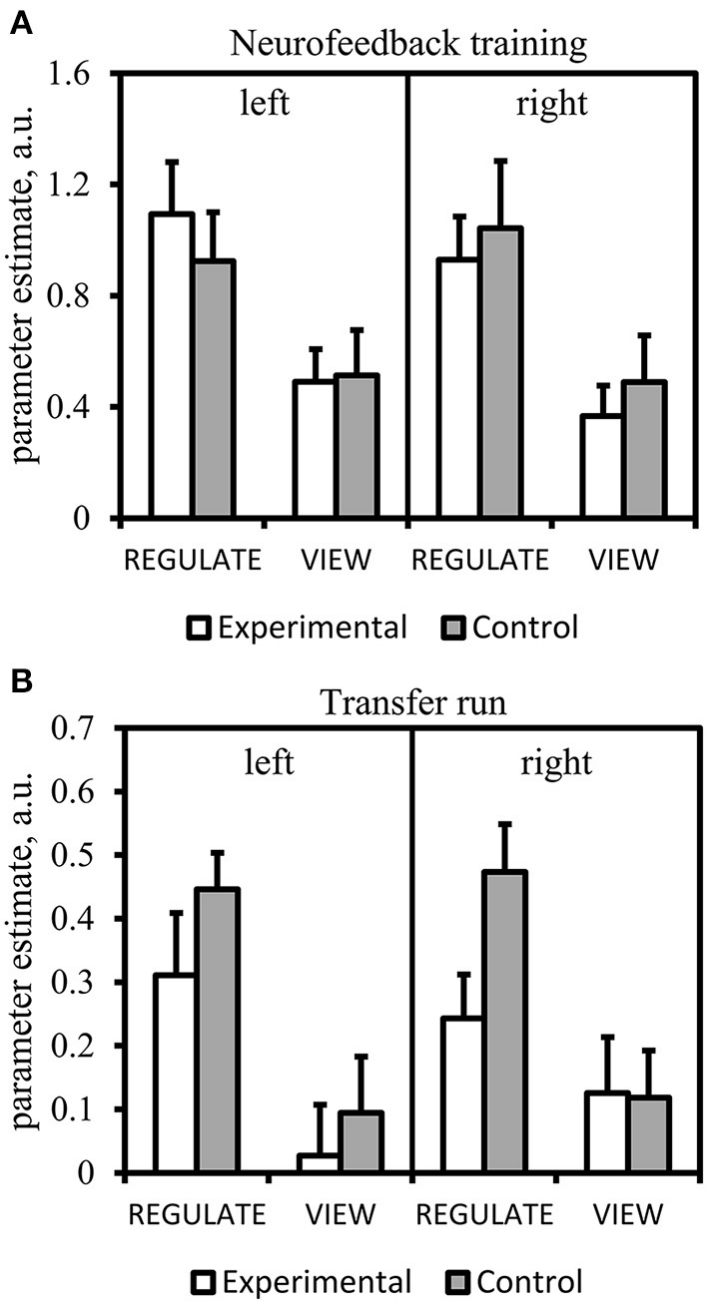
**Results of the region-of-interest analysis of the anterior insula**. Mean parameter estimate of all voxels within the anterior insula masks of the REGULATE>NEUTRAL and VIEW>NEUTRAL contrast. Error bars indicate standard error of mean. A.u., artificial units. **(A)** Neurofeedback training (mean over all runs). **(B)** Transfer run.

Results from the Run × Condition × Group ANOVA can be obtained from Table 3A. There was a significant main effect of Condition with higher anterior insula activation during REGULATE vs. VIEW (Figure 6A). None of the interactions were significant. The Run × Group interaction was found at trend level. A linear decrease of anterior insula activation, however, was not visible from the data of both groups.

**Table 3:** **Group statistics and ANOVA results of the anterior insula analysis**.

**(A)**		**Neurofeedback training**					
	**Hemisphere:**	**Left**			**Right**		
	**Run:**	**1**	**2**	**3**	**1**	**2**	**3**
**GROUP STATISTICS (MEAN ± *SD*)**							
Experimental group	REG>NEU	0.40 ± 0.35	0.35 ± 0.30	0.34 ± 0.34	0.30 ± 0.23	0.36 ± 0.25	0.27 ± 0.28
(*N* = 16)	VIEW>NEU	0.23 ± 0.24	0.08 ± 0.21	0.18 ± 0.28	0.15 ± 0.16	0.08 ± 0.26	0.14 ± 0.20
Control group	REG>NEU	0.43 ± 0.32	0.28 ± 0.39	0.22 ± 0.33	0.48 ± 0.43	0.15 ± 0.37	0.42 ± 0.47
(*N* = 16)	VIEW>NEU	0.24 ± 0.33	0.10 ± 0.43	0.17 ± 0.32	0.24 ± 0.40	0.00 ± 0.41	0.25 ± 0.37
**ANOVA RESULTS (*P*-VALUES)**							
Interaction effects	Run × condition × group	0.660			0.285		
	Condition × group	0.465			0.972		
	Run × group	0.841			0.072		
	Run × condition	0.257			0.601		
Main effects	Group	0.714			0.595		
	Run	0.164			0.067		
	Condition	0.000			0.000		
**(B)**		**Transfer run**					
	**Hemisphere:**	**Left**			**Right**		
**Group statistics (mean ± *SD*)**							
Experimental group	REG>NEU	0.31 ± 0.39			0.24 ± 0.28		
(*N* = 16)	VIEW>NEU	0.03 ± 0.32			0.13 ± 0.35		
Control group	REG>NEU	0.45 ± 0.23			0.47 ± 0.30		
(*N* = 16)	VIEW>NEU	0.09 ± 0.36			0.12 ± 0.30		
**ANOVA RESULTS (*P*-VALUES)**							
Interaction effect	Condition × group	0.593			0.095		
Main effects	Group	0.333			0.219		
	Condition	0.000			0.002		
*Post-hoc t*-test (*p*-value, two-tailed)	REG>NEU: control vs. experimental	0.258			0.037		
	VIEW>NEU: control vs. experimental	0.592			0.954		
	Experimental group: REG vs. VIEW	0.010			0.270		
	Control group: REG vs. VIEW	0.001			0.001		

*Table shows parameter-estimate-contrast values from the anterior insula spheres. **(A)** Neurofeedback training, **(B)** Transfer run. SD, standard deviation; REG, regulate; NEU, neutral*.

Table 3B lists the results of the transfer run. The Condition × Group effect was found at trend-level. The main effect of Condition was significant with higher anterior insula activation in the REGULATE vs. the VIEW condition. *Post-hoc t*-tests paralleled the findings from the amygdala ROI analysis: the experimental group showed less right anterior insula activation compared to the control group in the REGULATE condition. This difference was not present in the VIEW condition. Increased activation in the REGULATE vs. VIEW contrast was revealed in the bilateral anterior insula in the control group. The experimental group, however, did not significantly increase right anterior insula activation (Figure 6B).

### Exploratory whole-brain analysis

#### Neurofeedback training

No significant group comparisons were detected with a whole-brain analysis. When inspecting brain activation associated with natural responding to aversive picture viewing (VIEW>NEUTRAL), the experimental (Supplementary Table 1A) and the control group (Supplementary Table 1B) showed a similar pattern of activated brain regions, including the occipital lobe, the inferior temporal lobe and medial temporal areas. Activations were also found in the ventrolateral cortex, extending to the anterior insula. An analysis of brain areas implicated in regulation (REGULATE>NEUTRAL) brought only one significant cluster for the experimental group in the medial parietal lobe. In contrast, the analysis of the control group showed activated clusters covering occipital, parietal, inferior temporal, and prefontal areas. Activations were also detected in the anterior insula and in the medial temporal lobe. The analysis of brain regions with reduced responding during regulation with amygdala NF (VIEW>REGULATE) brought an activation cluster in the ventromedial PFC and another one in the medial occipital/temporal lobes. In contrast, no significant clusters were detected for the control group. The inspection of areas activated by regulation compared to natural responding (REGULATE>VIEW) in the experimental group brought one significant cluster in the right dorsolateral PFC. The control group, in contrast, activated an area in the dorsomedial frontal cortex and the thalamus.

#### Transfer run

Again, no group differences were found for neither of the contrasts. Supplementary Table 2 summarizes the findings of the single-group analyses. Taken together, activations were largely congruent in the VIEW>NEUTRAL contrast. Brain regions involved were found in the occipital lobe, inferior temporal lobe (including the fusiform gyrus) and medial temporal lobe (including the bilateral amygdala). At the whole-brain, both groups also responded similar when instructed to regulate (REGULATE>NEUTRAL). Besides activation clusters spanning the occipital lobe and inferior temporal lobe, both groups activated the dorsomedial frontal cortex and the dorsolateral PFC. Consistent with the ROI analysis, the experimental group showed activation in the left but not right amygdala in this contrast. No activations were found in the VIEW>REGULATE contrast. While control participants activated clusters in the dorsomedial frontal cortex, anterior insula and left ventrolateral PFC contrasting REGULATE vs. VIEW, no activations exceeded the cluster-threshold in the experimental group.

### Rating data

A repeated measures ANOVA for arousal and valence ratings of the stimuli used in the training neither showed significant group differences nor within-subjects effects (VIEW vs. REGULATE). There was also no significant Group interaction or main effect of Group found. An analysis of the arousal and valence ratings of the stimuli used in the transfer run also did not show significant group or within-subjects effects.

In terms of perceived regulation success, we did not find any significant differences between the experimental group [3.04 ± 1.89 (mean ± *SD*)] and the control group [3.85 ± 1.82; *T*_(30)_ = 1.530, *p* = 0.226]. Results did not change significantly when participants' ratings were compared on a run-by-run basis.

### Thermometer variability

The three-way ANOVA did not indicate an effect of Group on the variance of the number of bars displayed during the experimental runs (interactions: Condition × Run × Group: *p* = 0.949, Run × Group: *p* = 0.149, Condition × Group: *p* = 0.582; main effect of Group: *p* = 0.685).

## Discussion

Within one session of rt-fMRI-NF training, subjects receiving contingent amygdala feedback were successful in down-regulating amygdala activation in response to aversive pictures. In a subsequent run, subjects were able to decrease right amygdala activation without the feedback signal, which is in line with the hypothesis of a transfer of learned amygdala down-regulation. A control group receiving feedback from a standardized control region located in the rostal caudate did also reduce amygdala activation during training. A significant Region by Hemisphere by Group interaction indicated a specific effect of rt-fMRI-NF training on brain self-regulation in the transfer test. A trend in the two-way interactions of the amygdala (Hemisphere by Group) and right hemisphere (Region by Group) may suggest that this effect was lateralized to the right side. This proof of concept study is in line with previous reports on rt-fMRI-NF training of amygdala regulation (Zotev et al., 2011; Brühl et al., 2014) and extends the existing literature by providing evidence for the feasibility to use affective pictures as stimulus material to train amygdala down-regulation.

To provide evidence for a specificity of the treatment, the effect of the intervention needs to exceed the effect of a sham treatment which mimics external aspects of the real treatment. In other words, a placebo control is needed. In our study, we used the signal of a spatially well-defined control region located at the rostral caudate. We chose this region for its similarity with the experimental region in tissue composition and we matched the number of voxels taken to extract the NF signal for both groups. Furthermore, the literature does not indicate a function of the caudate comparable to the amygdala in emotion processing. This suggests that feedback from the control region may not specifically help in down-regulating amygdala activation in response to aversive stimuli. *Post-hoc* tests of the training phase illustrate, that the control region showed a positive change in BOLD signal amplitude in response to aversive pictures during REGULATE and VIEW (Supplementary Figure 1) and an analysis on the dynamics of the thermometer display does not indicate a significant difference between groups. The belief of receiving contingent feedback of affective brain activation may have prevented control subjects to become suspicious of a sham treatment or to become frustrated because of failure during the course of training. Due to the instructions, subjects knew that they would somehow have to engage in emotion regulation. Since we tested healthy participants, it is conceivable that all subjects were equipped with effective strategies to control their emotions in everyday life, and they may have employed these strategies to try to regulate the thermometer during training. Subjects of both groups may have applied strategies they had evaluated as successful based on their interpretation of the feedback signal and this may have impeded the detection of significant group differences during training. In this context it is not surprising that ratings of regulation success do not indicate a group difference. However, regulation-success was only estimated to be in the medium to low range. The lack of a difference in subjective success is in line with a previous report by Lawrence et al. (2013), who had trained one group with anterior insula NF and a control group with feedback from a control region located in the parietal lobe. It may be that a retrospective evaluation of regulation success is not sensible to capture between-group differences, especially when using placebo-feedback as a control. A better way to assess group differences may be via behavioral measures which are associated with the specific psychological function the intervention is thought to modulate. Regarding amygdala NF, future studies could let participants rate the subjective arousal and valence after regulation vs. natural viewing, which is known to be associated with amygdala activation to affective pictures (Zald, 2003; Anders et al., 2004).

The results of the transfer run provide first evidence that a rt-fMRI-NF training of amygdala down-regulation can have a differential effect on brain self-regulation in a placebo-controlled study. Further, results indicate a hemispheric asymmetry of brain self-regulation. There is an ongoing debate on a functional differentiation between left and right amygdala (Baas et al., 2004; Costafreda et al., 2008; Sergerie et al., 2008). However, methodological issues have been raised on interpreting laterality differences (LaBar et al., 2001; Mathiak et al., 2012) and no consistent laterality difference of amygdala activation has been reported in the emotion regulation literature (Buhle et al., 2013). A possible but rather speculative explanation for a lateralized transfer effect may state that subjects had tried different emotion regulation strategies during training which may have been successful in down-regulating the right as well as left amygdala. In the transfer run, however, participants may have used a strategy they had evaluated during training as most successful. Strategies approved as most successful may have preferentially involved neural processes of top-down control of the right amygdala. This interpretation endorses the choice of Brühl et al. (2014) to use the right amygdala for NF training.

The results of the anterior insula analysis largely complemented the results of the amygdala analysis. In the transfer run, the experimental group did not show a significant difference of right anterior insula responding when comparing the REGULATE to the VIEW condition, while the control group significantly up-regulated the anterior insula when instructed to regulate. In the group comparison, the experimental group showed less activation during REGULATE than the control group, which is in support of improved regulation of affective brain structures after amygdala NF. The group interaction, however, did only show a trend. As opposed to a training effect on the regulation of a rather circumscribed component of the brain's affective system, this result suggests that amygdala NF training may extend to the self-regulation of other brain regions in the domain of emotional processing. Future studies are needed to corroborate this finding.

To evaluate general effects of training and transfer, we conducted a whole-brain analysis between and within groups. The between-group analysis did not show significant group interactions. When instructed to naturally respond to aversive picture presentation, both groups congruently activated regions of the medial temporal lobe and the anterior insula, which are consistently found to be involved in the processing of emotion stimuli (Phan et al., 2002; Kober et al., 2008). This finding was consistent across the training and the transfer phase. When contrasting the REGULATE-condition against the non-affective control condition, both groups showed highly overlapping brain regions in the analysis of the transfer run. Results indicate that subjects from both groups had engaged in the top-down control of limbic and para-limbic regions when instructed to regulate their brain responses to aversive stimuli after training. The involvement of lateral PFC (Kalisch, 2009; Buhle et al., 2013), medial PFC/anterior cingulate cortex (Etkin et al., 2011; Paret et al., 2011), and anterior insula (Dosenbach et al., 2006; Hollmann et al., 2012; Veit et al., 2012) in emotion regulation and cognitive control is consistent with the existing fMRI literature. The single-group analyses of the REGULATE condition differed, however, regarding the rt-fMRI-NF training. Contrasting regulation with natural viewing of aversive pictures in the experimental group brought an acitivation cluster in the right lateral PFC, while the control group displayed activation of the dorsomedial frontal cortex. In the transfer run, the dorsomedial frontal cortex was again found activated only by the control group, indicating the involvement of similar brain structures during training and transfer in this group. The results may give a hint for the neural patterns implicated in down-regulation of the amygdala with and without contingent amygdala NF training. However, between-group differences may result from voxel-thresholding and may not reflect real differences in the involvement of brain regions between groups. Since we did not find significant group interactions, between-group differences remain descriptive and should be interpreted with caution.

We did not find evidence for a run-to-run improvement of amygdala down-regulation in the experimental group. This could have had several reasons, like exhaustion due to the duration of the scanning session or task difficulty. Future studies should try to improve the task design in order to make the NF training more efficient.

There are several limitations for this study. Regarding our hypotheses, we cannot conclude from the results that rt-fMRI-NF had a specific effect on the down-regulation of the BOLD signal amplitude of the amygdala response during the training phase. Additionally, we did not include a pre-training test to evaluate pre-post comparisons in amygdala regulation success. However, without any experience of rt-fMRI-NF, the instruction to regulate a brain region (the “feeling center”) may be difficult to follow. Therefore, and to prevent exhaustion due to long scanning duration, we decided against a run similar to the transfer run before the training. Data from pre-post comparisons, however, could advance our understanding of whether and to what extent amygdala NF training can change the activation of neural circuitries of emotion regulation and may be easier to implement with another experimental design. Another potential limitation may be the display of contingent feedback during the control conditions (VIEW and NEUTRAL). First, subjects of both groups reported that the thermometer did sometimes rise during the NEUTRAL condition. Signal increases without an external aversive stimulus may have had several reasons, such as affective responses to internal stimuli or signal noise. This may have confused participants and could have made them suspicious of receiving a sham feedback. Second, contingent amygdala feedback during VIEW may have triggered evaluation and control processes similar as in the REGULATE condition. An at-trend main effect of the factor Group in the Condition × Group ANOVA of the right amygdala is in line with this interpretation and may result from the generalization of learned regulation to the natural viewing condition. Future studies are needed for replication and should consider the potential confound of a feedback display during the control condition in the study design. In this study only healthy female subjects were tested. There are fMRI studies reporting gender differences in the involvement of prefrontal and limbic regions during emotion regulation (McRae et al., 2008; Mak et al., 2009; Domes et al., 2010). Thus, the results reported here may not generalize to samples of male participants.

In conclusion, this study shows that contingent rt-fMRI-NF from the amygdala may improve the regulation of amygdala activation in response to aversive pictures. A test of transfer effects showed an influence of amygdala feedback vs. feedback from a control region on brain self-regulation. This study is a starting point for further research toward the application of rt-fMRI-NF of the amygdala as a potential intervention in psychiatric populations. In particular, down-regulation of the amygdala as demonstrated in the current study and elsewhere (Brühl et al., 2014) may be helpful for disorders characterized by problems in emotion regulation and elevated amygdala activity such as borderline personality disorder. In these patients, training skills for emotion regulation is a decisive aspect of successful psychotherapies (Stoffers et al., 2012). NF may be used to test and train individual emotion regulation skills and therefore provide an excellent tool to increase efficacy and time-to-success of psychotherapy in such conditions.

## Author contributions

Christian Paret, Rosemarie Kluetsch, Matthias Ruf, Traute Demirakca, Gabriele Ende, and Christian Schmahl were involved in the design and conception of the work. Rosemarie Kluetsch, Steffen Hoesterey, and Christian Paret conducted the data acquisition. Christian Paret analyzed the fMRI data and wrote the manuscript. All authors (Christian Paret, Rosemarie Kluetsch, Matthias Ruf, Traute Demirakca, Steffen Hoesterey, Gabriele Ende, Christian Schmahl) were involved in the interpretation of data and revised the draft critically for important intellectual content. All authors approved the final version to be published and agreed to be accountable for all aspects of the work.

### Conflict of interest statement

The authors declare that the research was conducted in the absence of any commercial or financial relationships that could be construed as a potential conflict of interest.
