# Supplementary material for: Down-regulation of amygdala activation with real-time fMRI neurofeedback in a healthy female sample
Source: Front Behav Neurosci. 2014 Sep 18;8:299. doi: 10.3389/fnbeh.2014.00299 (PMC4166899; doi:10.3389/fnbeh.2014.00299)
Supplement: Supplementary file 1 [file Presentation1.PDF]

Supplementary Material (Paret et al., Down-regulation of the amygdala with real-time fMRI neurofeedback in a healthy female sample):

Supplementary Table 1A. Experimental group: exploratory whole-brain analysis of real-time fMRI neurofeedback training.

A) Experimental group

**VIEW>REG**	**brain region**	**hemisphere**	**k**	**Z-score**	**MNI (x, y, z)**
kmin=41	parahippocampal gyrus/ anterior calcarine sulcus, extending to:	left	217	4.58	-15,-55,10
	precuneus	right		3.85	6,-61,22
	precuneus	left		3.71	-6,-58,19
	medial frontal gyrus/ orbitofrontal cortex, extending to:	left	75	4.2	-6,65,-11
	medial frontal gyrus	right		4.15	9,56,-14
	medial frontal gyrus	left		4.07	-9,56,-14

**REG>VIEW**	**brain region**	**hemisphere**	**k**	**Z-score**	**MNI (x, y, z)**
kmin=41	middle frontal gyrus, extending to:	right	68	4.51	39,56,19
	middle frontal gyrus	right		3.78	36,50,25
	middle frontal gyrus	right		3.18	33,53,34

**REG>NEU**	**brain region**	**hemisphere**	**k**	**Z-score**	**MNI (x, y, z)**
kmin=42	angular gyrus, extending to:	left	52	3.94	-42,-64,34
	superior parietal lobe	left		3.77	-36,-70,46
	superior parietal lobe	left		3.57	-42,-76,43

**VIEW>NEU**	**brain region**	**hemisphere**	**k**	**Z-score**	**MNI (x, y, z)**
kmin=37	thalamus, extending to:	right	12328	6.28	18,-31,1
	occipital cortex, ext. to parietal cortex	left		6.19	-39,-85,-2
	occipital cortex, ext. to parietal cortex	right		6.14	27,-55,-5
	thalamus	left		6.06	-18,-31,4
	amygdala	right		5.8	27,-4,-20
	amygdala	left		5.66	-18,-7,-17
	middle frontal gyrus, extending to:	right	63	4.73	36,2,52
	middle frontal gyrus			3.82	36,-1,64
	medial frontal gyrus, extending to:	right	625	4.7	15,50,52
	medial frontal gyrus			4.66	6,59,28
	medial frontal gyrus			4.5	12,56,43
	orbitofrontal cortex/medial frontal gyrus, extending to:	right	297	4.58	6,44,-26
	medial frontal gyrus			4.51	0,62,-11
	medial frontal gyrus			4.4	0,44,-20

SMA/superior frontal gyrus, extending to:	right	82	4.12	9,14,67
SMA/medial frontal gyrus	left		3.67	-6,11,58
SMA/medial frontal gyrus	right		3.61	9,14,49
inferior temporal gyrus, extending to:	right	63	4.04	60,-1,-35
inferior temporal gyrus	right		3.69	66,-7,-26
superior temporal gyrus	right		3.67	54,-4,-20
middle frontal gyrus, extending to:	left	50	3.98	-39,8,28
middle frontal gyrus	left		3.6	-45,-1,40
middle frontal gyrus	left		3.55	-36,17,25

B) Control group

**VIEW>REG**	**brain region**	**hemisphere**	**k**	**Z-score**	**MNI (x, y, z)**
kmin=53	*no activations found*				
kmin=41	*no activations found*				

**REG>VIEW**	**brain region**	**hemisphere**	**k**	**Z-score**	**MNI (x, y, z)**
kmin=53	medial frontal gyrus, extending to:	right	101	4.66	6,23,43
	medial frontal gyrus	right		3.27	3,5,58
	medial frontal gyrus	right		3.16	15,23,52
	superior frontal gyrus, extending to:	right	87	4.25	18,8,64
	superior frontal gyrus	right		3.66	30,8,67
	thalamus, extending to:	right	78	4.15	15,-13,16
	thalamus	right		3.95	6,-7,4
	thalamus	left		3.66	-6,-13,10

**REG>NEU**	**brain region**	**hemisphere**	**k**	**Z-score**	**MNI (x, y, z)**
kmin=46	middle occipital lobe, extending to:	left	7187	6.55	-39,-82,-2
	occipital lobe	right		6.54	42,-79,4
	occipitotemporal gyrus	right		6.09	42,-40,-20
	brain stem	right		5.91	9,-25,-11
	occipitotemporal gyrus	left		5.86	-36,-49,-20
	uncus	left		5.10	-21,-7,-14
	uncus	right		5.01	21,-4,-11
	anterior insula	left		4.8	-30,20,-8
	antirior insula	right		4.15	39,29,1
	precentral gyrus, extending to:	right	488	5.61	39,11,25
	middle frontal gyrus	right		4.26	45,2,58
	middle frontal gyrus	right		4.26	48,8,37
	medial frontal gyrus, extending	medial	311	5.45	0,14,46

	to:				
	SMA/superior frontal gyrus	right		5.28	15,11,67
	medial frontal gyrus	left		3.87	-9,8,58
	middle frontal gyrus, extending to:	left	217	4.64	-39,8,28
	precentral gyrus	left		4.25	-42,-4,55
	precentral gyrus	left		4.1	-39,-1,40
	superior parietal lobe/ precuneus, extending to:	right	76	4.2	9,-49,52
	postcentral gyrus	medial		4.13	0,-52,55
	superior parietal lobe	right		4.02	6,64,61

**VIEW>NEU**	**brain region**	**hemisphere**	**k**	**Z-score**	**MNI (x, y, z)**
kmin=43	inferior occipital gyrus, extending to:	left	7389	7.24	-42,-79,-2
	occipital gyrus	right		6.59	39,-64,-11
	occipitotemporal gyrus	left		6.43	-36,-49,-20
	occipitotemporal gyrus	right		6.36	39,-70,-14
	inferior orbital gyrus	left		6.03	-36,26,-14
	hippocampus/uncus	left		5.98	-30,-7,-17
	amygdala	left		4.76	-24,-1,-17
	amygdala	right		4.73	33,-1,-17
	amterior insula	left		4.55	-39,23,1
	superior medial frontal gyrus, extending to:	left	263	5.64	-9,62,34
	superior medial frontal	right		4.59	6,62,37
	superior medial frontal	right		4.51	9,68,22
	orbitofrontal cortex/medial frontal gyrus, extending to:	left	167	5.15	-3,50,-14
	medial frontal gyrus	right		4.94	3,41,-17
	inferior frontal gyrus/ anterior insula, extending to:	right	62	4.52	45,26,-2
	inferior frontal gyrus	right		3.92	51,29,-8
	middle frontal gyrus, extending to:	right	89	4.23	48,32,25
	middle frontal gyrus	right		3.97	54,20,16
	middle frontal gyrus	right		3.81	57,29,25

Table lists results from the whole-brain analysis of each contrast. The voxel peak-threshold was set at $p<0.001$ (uncorrected) and the minimum cluster size (kmin) was adjusted to protect against false positives with Monte-Carlo simulations (cluster-threshold at $p<0.05$). The VIEW>REG analysis was repeated once for the control group with the same cluster-extent threshold determined for the experimental group for reasons of comparability. Table lists coordinates and test-values of peak voxels in activated clusters. X, y and z coordinates are based on the MNI-coordinate system. Cluster-peaks listed are at least 8 mm apart. K=cluster size, MNI=Montreal Neurological Institute, REG=REGULATE, NEU=NEUTRAL.

Supplementary Table 2. Exploratory whole-brain analysis of the transfer run.

A) Experimental group

**VIEW>REG**	**brain region**	**hemisphere**	**k**	**Z-score**	**MNI (x, y, z)**
kmin=40	*no activations found*				

**REG>VIEW**	**brain region**	**hemisphere**	**k**	**Z-score**	**MNI (x, y, z)**
kmin=40	*no activations found*				

**REG>NEU**	**brain region**	**hemisphere**	**k**	**Z-score**	**MNI (x, y, z)**
kmin=42	middle temporal gyrus, extending to:	right	1392	5.96	48,-70,4
	middle temporal gyrus	right		5.52	57,-61,13
	inferior temporal lobe	right		5.36	39,-52,-14
	middle temporal gyrus, extending to:	left	1028	5.34	-42,-61,13
	middle temporal gyrus	left		4.91	-45,-70,16
	middle frontal gyrus, extending to:	left	167	4.89	-39,11,28
	inferior frontal gyrus	left		4.02	-54,23,13
	inferior frontal gyrus	left		3.82	-48,17,4
	middle frontal gyrus, extending to:	right	805	4.67	42,8,37
	inferior frontal gyrus	right		4.50	48,26,19
	SMA/superior frontal	right		4.38	12,11,61
	medial frontal gyrus/cingulum	right		3.88	9,14,43
	middle frontal gyrus, extending to:	left	66	4.52	-39,-1,52
	middle frontal gyrus	left		4.01	-48,2,46
	middle frontal gyrus	left		3.81	-48,8,55
	supramarginal gyrus, extending to:	left	48	3.86	-66,-37,31
	supramarginal gyrus	left		3.82	-57,-43,31
	supramarginal gyrus	left		3.48	-57,-49,22
	uncus/superior temporal pole, extending to:	left	49	3.79	-30,8,-23
	amygdala	left		3.65	-30,-1,-26
	amygdala	left		3.63	-21,-7,-17

**VIEW>NEU**	**brain region**	**hemisphere**	**k**	**Z-score**	**MNI (x, y, z)**
kmin=39	inferior occipital gyrus, extending to:	right	1740	5.34	45,-76,-2
	middle temporal gyrus	right		5.24	45,-70,13
	fusiform/occipitotemporal gyrus	right		5.17	39,-40,-26
	inferior occipital gyrus, extending to:	left	1141	5.16	-42,-82,-2
	middle occipital gyrus	left		4.97	-45,-61,13

	inferior temporal gyrus	left		4.83	-42,-43,-14
	amygdala, extending to:	left	54	4.64	-24,-4,-20
	amygdala	left		4.15	-33,-4,-17
	amygdala	left		3.35	-18,-4,-11
	inferior frontal gyrus, extending to:	right	50	4.61	60,29,22
	inferior frontal gyrus	right		3.91	54,26,13
	inferior frontal gyrus	right		3.65	54,35,10
	amygdala, extending to:	right	47	3.95	27,-1,-20
	amygdala	right		3.61	18,-7,-11

B) Control group

VIEW>REG	brain region	hemisphere	k	Z-score	MNI (x, y, z)
kmin=46	*no activations found*				

REG>VIEW	brain region	hemisphere	k	Z-score	MNI (x, y, z)
kmin=46	anterior insula, extending to:	right	160	4.59	39,20,-5
	inferior frontal gyrus	right		3.75	51,20,10
	anterior insula	right		3.67	33,29,-2
	inferior frontal gyrus, extending to:	left	47	4.11	-51,47,-5
	inferior frontal gyrus	left		4.08	-42,41,1
	inferior frontal gyrus	left		3.54	-57,35,1
	medial frontal gyrus/cingulum, extending to:	right	84	3.91	6,26,40
	medial frontal/cingulum	medial		3.57	0,17,43

REG>NEU	brain region	hemisphere	k	Z-score	MNI (x, y, z)
kmin=47	fusiform/occipitotemporal gyrus, extending to:	right	1836	6.29	39,-61,-14
	occipital pole	right		6.14	36,-85,4
	fusiform/occipitotemporal	right		6.12	36,-49,-23
	inferior occipital gyrus, extending to:	left	2068	6.26	-42,-82,-5
	inferior occipital gyrus	left		5.92	-48,-73,-5
	inferior occipital gyrus	left		5.82	-39,-73,-17
	middle frontal gyrus, extending to:	right	1054	5.91	48,23,28
	middle frontal gyrus	right		5.45	42,14,25
	anterior insula	right		5.24	33,26,1
	anterior insula, extending to:	left	510	5.25	-36,23,-2
	middle frontal gyrus	left		4.97	-54,17,1
	middle frontal gyrus	left		4.64	-60,17,16
	caudate nucleus, extending to:	right	123	4.37	18,-4,19
	caudate nucleus	right		4.12	15,8,10

	caudate nucleus	right		3.97	6,-10,7
	middle frontal gyrus, extending to:	left	234	4.23	-42,8,34
	middle frontal gyrus	left		4.03	-39,-1,48
	middle frontal gyrus	left		3.93	-45,17,28
	medial superior frontal gyrus, extending to:	medial	301	4.13	0,32,49
	superior frontal gyrus	right		4.01	9,8,70
	medial superior frontal gyrus	right		3.97	3,17,61
	superior parietal lobe, extending to:	right	55	3.84	33,-52,49
	superior parietal lobe	right		3.65	30,-58,61

**VIEW>NEU**	**brain region**	**hemisphere**	**k**	**Z-score**	**MNI (x, y, z)**
kmin=38	occipital lobe, extending to:	left	1708	6.49	-36,-85,-2
	occipital lobe	left		6.17	-51,-73,-8
	occipital lobe	left		5.99	-27,-82,-2
	fusiform/occipitotemporal gyrus	left		5.37	-36,-49,-20
	fusiform/occipitotemporal gyrus, extending to:	right	2212	6.16	42,-49,-20
	fusiform/occipitotemporal gyrus	right		5.94	39,-64,-11
	occipitotemporal lobe	right		5.88	42,-82,-2
	thalamus	right	118	5.18	18,-31,1
	thalamus	left	48	4.62	-18,-31,1
	hippocampus, extending to:	right	116	4.41	21,-7,-17
	amygdala	right		4.3	30,-4,-14
	hippocampus	right		3.72	33,-16,-17
	inferior frontal gyrus, extending to:	right	219	4.39	54,29,16
	inferior frontal gyrus	right		4.32	54,29,25
	inferior frontal gyrus	right		4.13	48,38,10
	amygdala/hippocampus	left	73	4.14	-21,-10,-17

Table lists results from the whole-brain analysis of each contrast. The voxel peak-threshold was set at $p<0.001$ (uncorrected) and the minimum cluster size (kmin) was adjusted to protect against false positives with Monte-Carlo simulations (cluster-threshold at $p<0.05$). Table lists coordinates and test-values of peak voxels in activated clusters. X, y and z coordinates are based on the MNI-coordinate system. Cluster-peaks listed are at least 8 mm apart. K=cluster size, MNI=Montreal Neurological Institute, REG=REGULATE, NEU=NEUTRAL.

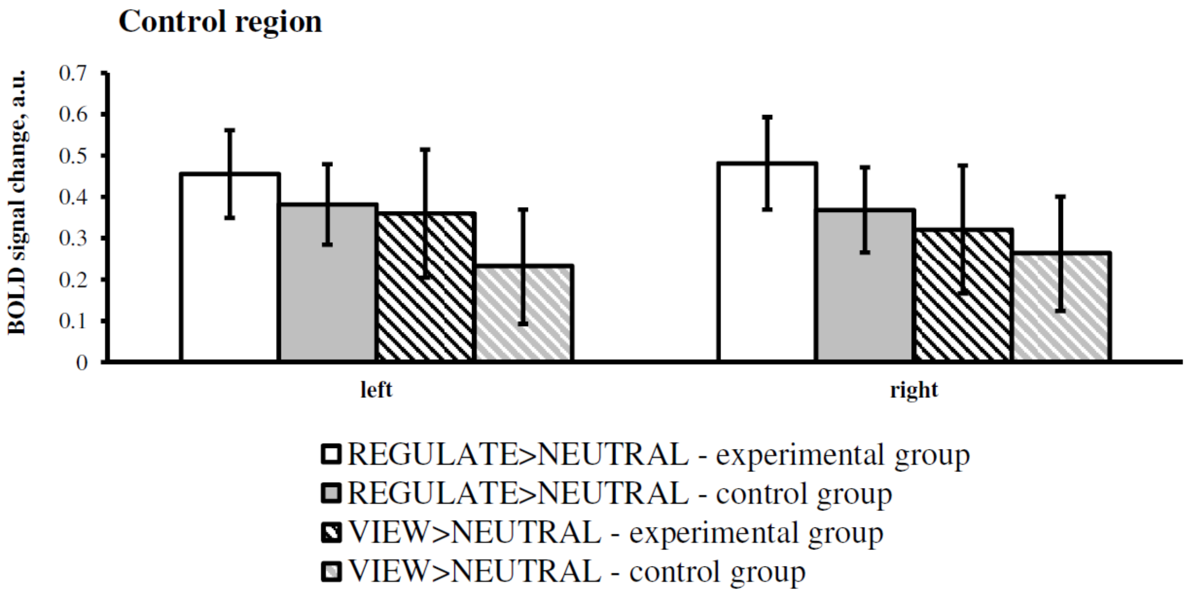

Supplementary Figure 1.Neurofeedback training. Group means of parameter estimates. Error bars indicate standard error of mean. Participants receiving feedback from a non-standardized control region were excluded for the preparation of this figure. BOLD=Blood Oxygenation Level Dependent, a.u.= aritificial units.
